# Different clearance of *KIT*D816V mutation and tryptase levels after haematopoietic cell transplantation in patients with systemic mastocytosis with associated haematological neoplasm

**DOI:** 10.1111/bjh.20211

**Published:** 2025-06-17

**Authors:** Christian Niederwieser, Anita Badbaran, Radwan Massoud, Nico Gagelmann, Ameya Kunte, Evgeny Klyuchnikov, Niloufar Seyedi, Silke Heidenreich, Ina Rudolph, Gaby Zeck, Catherina Lück, Dietlinde Janson, Christine Wolschke, Francis Ayuk, Nicolaus Kröger

**Affiliations:** ^1^ Department of Stem Cell Transplantation University Medical Center Hamburg‐Eppendorf Hamburg Germany

**Keywords:** allogeneic, HCT, *KIT*, reduced intensity conditioning, SM‐AHN, tryptase

## Abstract

The patterns of tryptase normalization, *KIT*D816V clearance and establishment of donor cell chimerism were analysed in 13 patients with systemic mastocytosis and associated haematological neoplasm (AHN) after haematopoietic cell transplantation (HCT). The molecular marker of systemic mastocytosis (SM) (*KIT*D816V) simultaneously disappeared (median + 36 days) with the establishment of donor chimerism (median: +31 days post‐HCT; Pearson correlation: −0.99, *p* < 0.001). In contrast, tryptase normalized median of +228 days after HCT with significant delay to *KIT*D816V clearance (*p* = 0.01) and full donor chimerism (*p* = 0.04). Faster normalization was observed after radiation‐based conditioning and removal of an infiltrated spleen, while persistence was not associated with relapse of AHN.

## INTRODUCTION

Systemic mastocytosis (SM) with associated haematological neoplasm (AHN) is a subgroup of SM^1^, ^2^ with complex neoplastic biology, pathology and variable clinical course. Patients with SM‐AHN suffer from symptoms caused by mast cells (MCs) expansion leading to bone marrow (BM) suppression and altered organ function like cytopenia, malabsorption, hepato‐splenomegaly or osteopenia in addition to AHN symptoms.^3^

SM‐AHN definition involves one major (multifocal aggregates of >15 MCs) and one minor (atypical morphology of MCs, *KIT*D816X, CD25^+^) or three minor criteria of SM. SM‐AHN has been classified in different types including indolent systemic mastocytosis‐AHN, aggressive systemic mastocytosis (ASM)‐AHN and mast cell leukaemia (MCL)‐AHN.^2^ Pathognomonic for the disease in >90% of patients are somatic gain‐of‐function mutations in *KIT*D816V mutation acting as a weak oncogene on proliferation but a strong inducer of MC differentiation, maturation and survival.^4^ Tryptase is a secretory granule‐derived serine proteinase of MC, a marker of MC activation and mastocytosis activity in general.

Overall survival (OS) of SM‐AHN amounts to a median of 2.9 years,^3^ but ranges according to the Mutation‐Adjusted Risk Score for Advanced Systemic Mastocytosis (MARS) score from few months to several years.^5^ Therapy should account for SM and AHN. Systemic cytotoxic treatment with the tyrosine kinase inhibitor midostaurin is considered the golden standard for SM.^6^ Therapy of AHN including haematopoietic cell transplantation (HCT) might be more relevant to the prognosis.

The replacement of the haematopoietic system in SM‐AHN patients represents a unique opportunity to understand pathophysiology and interactions of both diseases in more detail. Therefore, kinetics of SM markers like serum tryptase and *KIT*D816V mutation, haematopoietic engraftment (chimerism) after HCT and clinical complications were correlated.

## PATIENTS AND METHODS

Patients with SM‐AHN (*n* = 13; 0.3%) were identified out of >4200 HCT between 2006 and 2022 at the University Medical Center Hamburg. Disease and clinical characteristics of the patients are given in Table 1 and included diagnoses of SM‐myelodysplastic syndrome (MDS)/myeloproliferative neoplasm (*n* = 9), SM‐MDS (*n* = 1), SM‐primary myelofibrosis (PMF) (*n* = 1), SM‐acute myeloid leukaemia (AML) (*n* = 1) and SM‐chronic myeloid leukaemia accelerated phase (*n* = 1). SM type included MCL‐AHN in nine, ASM‐AHN in two and at least ASM‐AHN in a further two patients (Table 1; Table S1). A *KIT*D816V mutation (*n* = 12; one with additional *KIT*K558E) was present in all patients except patient no. 3 with *KIT*M541L. HCT (*n* = 14) was performed after reduced intensity (*n* = 8) or myeloablative preparative regimens (*n* = 6) from HLA compatible related/unrelated or haploidentical‐related (*n* = 3) donors. Peripheral blood (PB) was used as a graft source in 12 of 13 patients containing a median of 6.1 (range: 1.5–10.5) ×10^6^/kg CD34^+^ cells. Graft‐versus‐host disease (GvHD)^7^ prophylaxis consisted of ciclosporin or tacrolimus and MMF starting on day −1 or on day 5 dependent on in vivo T‐cell depletion with anti‐T‐lymphocyte globulin in unrelated or on post‐transplant cyclophosphamide in haploidentical HCT. GvHD was assessed according to published guidelines.^7^

**TABLE 1:** Baseline clinical and HCT characteristics of SM‐AHN patients.

Characteristics	Details	*n* or median (range)
Sex	Male/female	11/2
Age at HCT	Years	60 (53–83)
Disease at diagnosis	MDS/MPN/MDS	9/1
	PMF/AML/accelerated phase CML	1/1/1
Cytogenetics	46,XY or 46,XX	6
	47,XY, +8; 46,XY t(9;22)(q34;q11); 47, XY,+11	1/1/1
	Complex	1
	n.a.	3
SM type	ASM/at least ASM/MCL	2/2/9
Treatment for SM	Yes/no	7/6
AHN disease at HCT	MDS/MPN	7
	AML/CML	5/1
Stage of AHN	Untreated	4
	CR/PR/PR	1/1/2
	PD/NR	2/1
	Relapse	1
	AP2	1
	n.a.	1
Cytogenetics at HCT	46,XY or 46 XX	8
	48;XY,+8,+8; 47,XY, +8; 46,XY t(9;22)(q34;q11)	1/1/1
	Complex	1
	n.a.	1
*KIT* ^mut^ before or at HCT	*N*	13
Karnofsky index at HCT (%)	100/80/60/n.a.	3/8/1/2
HCT/patients	*n*	14/13
Donor	Age	34 (23–62)
	Male/female	10/4
	Unrelated/related	10/4
	Match (10/10)/mismatch/haploidentical	8/3/3
Graft source	PBSC/BM/n.a.	12/1/n.a.
Diagnosis‐HCT interval	Years	1,9 (0,4‐3,7)
Preparative regimen	FLAMSA ± other/BuCY/BuTT/BuFlu ± other TreoFlu/TBI (8Gy) Flu	4/1/2/4/ 2/1
	RIC/MAC	8/6
GvHD prophylaxis	CSA/MMF, TAC/MMF, postCy	9/2/3
Chimerism (maximum)	%	99.9 (98.9–99.9)
GvHD acute	Grade 0/1/2/3/4	5/2/5/2/0
GvHD chronic	None/mild/moderate/severe/n.a.	5/5/3/0/1
AHN relapse	Yes/no	4/10
SM relapse	Yes/no	1/13

Abbreviations: AHN, associated haematological neoplasia; AML, acute myeloid leukaemia; AP2, accelerated phase 2; ASM, aggressive SM; BM, bone marrow; Bu, busulfan; CML, chronic myeloid leukaemia; CR, complete remission; CSA, ciclosporin; CY, cyclophosphamide; FLAMSA, Fludarabine/Amsacrine; Flu., fludarabine; GvHD, graft‐versus‐host disease; Gy, Gray; HCT, haematopoietic cell transplantation; MAC, myeloablative conditioning; MCL, mast cell leukaemia; NIH overall grade; MDS, myelodysplastic syndrome; MMF, mycofenolate mofetil; MPN, myeloproliferative neoplasm; n.a., not available; NR, non‐response; PBSC, peripheral blood stem cells; PD, progressive disease; PMF, primary myelofibrosis; postCy, post‐transplant‐cyclophosphamide; PR, partial remission; RIC, reduced intensity conditioning; SM, systemic mastocytosis; TAC, tacrolimus; TBI, total body irradiation; Treo, treosulfan; TT, thiotepa.

*KIT*D816V mutation, chimerism and tryptase were determined in BM on days 30, 100, 365 post‐HCT. In addition, *KIT*D816V mutation and chimerism were measured in PB at least every 2 months and annually after day 365. Mutation analyses were based on amplicon next‐generation sequencing (NGS) on DNA from PB using myeloid, AML, MDS or PMF panels and droplet digital (dd)PCR.^8^ Sensitivity was 3% for NGS and 0.10% for ddPCR (Bio‐Rad, article number dHsaMDV2010023). Tryptase was determined by ELISA with a normal value < 20 μg/L (ImmunoCAP, Thermo Fisher Scientific, Waltham, MA, USA) and, except in patient no. 13, maximum value at 200 μg/L. Whole chimerism was evaluated by PCR technique as recently reported.^9^

## STATISTICAL METHODS

OS (time from HCT to death) and disease‐free survival (DFS; time from HCT to haematological disease recurrence or death) were estimated by the Kaplan–Meier method. Relapse incidence (RI) and non‐relapse mortality (NRM) were analysed as mutually competing risks. The first HCT of patient no. 7 was censored at the time of the second HCT. Correlation studies between chimerism, *KIT*D816V mutation and tryptase were performed using the Pearson correlation (PC) test with the SPSS (version 29.0.0.0 (241) IBM, Armonk, NY). Time to normalization was determined by Kruskal–Wallis and *p*‐values <0.05 were considered statistically significant.

## RESULTS

**FIGURE 1:**
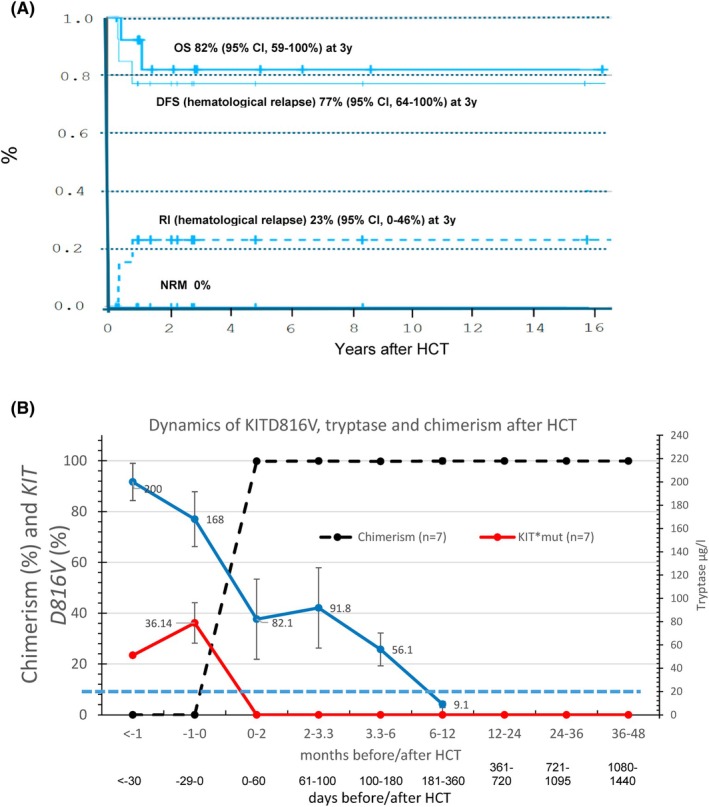
(A) Overall survival (OS), disease free survival (DFS), relapse incidence (RI) and non‐relapse mortality (NRM) of 14 haematopoietic cell transplantations (HCTs) in 13 patients with systemic mastoyctosis (SM)‐associated haematological neoplasia (AHN) (median follow‐up: 26.4 (3.4–189.2) months). (B) Total donor chimerism (median ± standard error %), *KIT*D816V (median ± standard error %) in blood and bone marrow cells and tryptase level (median ± standard error μg/L) in patients with SM‐AHN after HCT. The blue dotted line is the cut‐off value of tryptase.

After a median follow‐up of 26.4 (3.4–189.2) months, OS resulted in 82% (95% CI: 59%–100%), DFS 77% (95% CI: 64%–100%) and RI 23% (95% CI: 0%–46%) at 3 years without NRM (Figure 1A). There were no significant differences in the number of patients alive and with AHN relapses diagnosed with ASM‐AHN versus MCL‐AHN and after reduced intensity conditioning (RIC) versus myeloablative conditioning (MAC) (Table S2; *p* = n.s.); however, there is a trend in patients alive with SM‐AML versus SM‐non‐AML. One patient went on to second HCT and is alive without relapse of SM‐AHN.

Chimerism analyses were available in all 13 patients and longitudinally *KIT*D816V mutation in 7 patients (8 HCT) for a total of 74 time points. Tryptase was measured in 11 patients on 125 time points and sequentially in 8 HCT of 7 patients. Six patients with insufficient longitudinal measurements (≤2 of each of the three parameters/patient) were excluded. Interplay of donor chimerism, presence of *KIT*D816V mutation and tryptase levels before/after HCT are presented in Figure 1B. No difference in PB and in BM was detected on chimerism % and *KIT*D816V%.

All patients finally engrafted with full donor chimerism 31 (median; range: 26–100) days after HCT (Table 1; Figure 1B). *KIT*D816V mutation turned undetectable (measurable residual disease ≤0.05%) within a median of 36 (range: 26–100) days post HCT and was strongly negatively correlated with donor chimerism (PC: −0.9993; *p* < 0.001). In contrast, blood tryptase levels had a weaker, but statistically significant correlation with donor chimerism (PC: −0.1847; *p* < 0.001) and *KIT*D816V mutation (PC: 0.1717; *p* < 0.001). Chimerism versus *KIT*D816V and tryptase for each patient is given in Figure S1A,B. Tryptase levels decreased but normalized (<20 μg/L) only after a median of 228 (57–719) days post‐HCT (Figure 1B). The time difference to normalize tryptase blood level and fail to detect *KIT*D816V mutation (*p* = 0.035) or to reach full donor chimerism was statistically significant (*p* = 0.012).

Interestingly, a second tryptase peak was observed around day +100 during GvHD‐prophylaxis tapering (Figure 1B; Figures S4–S6). Individual tryptase, *KIT*D816V mutation and chimerism dynamics of singular patients are shown in the supplement (Figures S2–S8). In one patient, the *JAK2* mutation disappeared simultaneously with the *KIT*D816V 1 month after HCT (Figure S3).

Other NGS‐derived disease‐related mutations disappeared in 15 instances (*BRAF*, *SRSF2* (*n* = 2), *RUNX1* (*n* = 4), *U2AF1* (*n* = 2), *ASXL1*, *TET2* (*n* = 3), *KRAS*, *EZH2*) in seven patients and in three of those without relapse or death. In one patient with relapse, mutations not present after HCT appeared: *IDH1*R132H VAF, *GATA2*W360R, *JAK2*V617F, *NF1F*103fs*7 and *ZRSR2*S447‐R448dup.

Tryptase normalization was observed in one patient 9 days after splenectomy of a massively enlarged and histological/molecularly proven *KIT*D816V MC infiltrated spleen (Figure S6; Table S1). A decrease in tryptase level 57 days post‐HCT was also observed in a patient after conditioning with total body irradiation (TBI; Figure S2, 2.HCT). Furthermore, an increase in tryptase levels was observed in the absence of haematological relapse (Figure S7) except in one patient after graft rejection/relapse and detection of the *KIT*D816V mutation (Figure S2, 1.HCT).

## DISCUSSION

This analysis confirms the excellent efficacy of HCT in the treatment of SM‐AHN and in eradicating molecular markers of SM and AHN. However, kinetic differences of the analysed markers were detected. The disappearance of the SM marker *KIT*D816V mutation and full donor chimerism were strongly correlated and observed within a median of +36 and +31 days after HCT, respectively, but elevated tryptase levels normalized significantly later a median of +228 days post‐HCT (*p* = 0.04; *p* = 0.01). The slow decrease paralleled the presence of MC infiltration in BM (Table S1). A second peak around day +100 in three patients could be associated with GvHD prophylaxis tapering. Remarkable normalization of elevated tryptase levels was noted within days after splenectomy of a *KIT*D816V MC infiltrated spleen, but persistent negative *KIT*D816V in PB and BM (Figure S6) and a few weeks after TBI (Figure S2). Increase of tryptase was not associated with an increase in *KIT*D816V (Figure S7). In three patients with AHN relapse, no SM relapse was observed. Only in one patient with rejection/relapse, SM and AHN reappeared simultaneously (Figure S2 first HCT).

Knowledge about the origin, phenotypes and functions of MCs has increased substantially over the past 50 years. MCs are now known to derive from multipotent haematopoietic progenitors, which, through a process of differentiation and maturation, form a unique haematopoietic lineage and reside in multiple organs.^10^, ^11^ The morphological dissimilarity from other haematopoietic cells and the development of a distinct group of neoplasms support this view. Our observation confirms not only the unique entity of MCs but also the extramedullary persistence of local *KIT*D816V MC months after HCT. To our knowledge, no information on organ‐selective *KIT*D816V MC has been described to date, and no resistance of MC to HCT preparative regimens leading to increased tryptase levels is known. Our results suggest that persistent increased tryptase levels may result from MC in the non‐BM compartment until final replacement or removal. Furthermore, MC seems to be very sensitive to irradiation as shown by the fastest decrease after conditioning with 800 cGy TBI. Reports of skin MC being more sensitive to irradiation than BM‐MC may support this assumption.^12^, ^13^

Our results of persistent tryptase levels or even increase on day 100 after HCT are in line with previous reports showing the appearance of donor‐derived MC only >6 months after HCT in BM and PB, and the long time needed for drug‐induced MC eradication.^14^, ^15^ No other explanations in patients with persistent engraftment for elevated tryptase levels like concomitant renal insufficiency, anaphylaxis, GvHD, infection or hereditary alpha tryptasemia were found to be responsible in our patient cohort. An extramedullary graft‐versus‐MC effect from the described tryptase kinetics seems inefficient.

From the clinical point of view, HCT was able to cure SM‐AHN by replacing a haematopoietic system and to remove genetic changes typical for SM with impressive OS, DFS without increased haematological RI and no NRM. These results compare favourably to results published in recent multicentre studies with lower median age (References S1 and S2). In our cohort, the curative treatment option of HCT in SM‐AHN was confirmed by a median follow‐up of more than 2 up to 16 years. In our study, SM‐AML as compared to SM‐non‐AML had a trend for lower OS (*p* = 0.09). Lethality in our cohort was observed in ASM‐ and MCL‐AML, but there was no difference in lethality after RIC or MAC.

In our study, we present for the first time a detailed longitudinal study with concurrent molecular chimerism and biomarker analysis. Fast normalization of tryptase after TBI should be evaluated in irradiation‐containing preparative regimens in future protocols. The importance of drug‐induced MC eradication pre‐ or post‐transplant must be placed into perspective of our findings considering the persistent high tryptase levels in the absence of *KIT*D816V and without an apparent clinical negative effect. One patient with negative *KIT*D816V but elevated tryptase level showed limited decrease after midostaurin post‐transplant treatment for side effects (Figure S7). Several patients (*n* = 7) were treated for SM pre‐ and post‐transplant. Evidence for increased GvHD was not observed, but careful histological diagnosis to separate GvHD from SM symptoms may be needed.

Our observations also show that AHN and SM behave differently during relapse after HCT. This might be pertinent to a differentiated clone with self‐renewal capacity, but not involving the AHN clone.

In conclusion, HCT is a very efficient treatment of SM‐AHN resulting in impressive outcomes. Molecular remissions and establishment of full donor chimerism were closely associated. Tryptase normalization lagged behind molecular *KIT* ^mut^ remissions and donor cell chimerism. Although these observations need to be validated in larger trials, tryptase persistence and increase after HCT were not associated with relapse of the AHN, should not lead to treatment intensification and may challenge the current available recommendations (Reference S3).

## AUTHOR CONTRIBUTIONS

CN, AB, RM, NG, AK, EK, NS, SH, IR, GZ, CL, DJ, CW, FA and NK performed the research. CN and NK designed the research study. CN, AB, NG, AK, EK, NS, SH, IR, GZ, CL, DJ, CW, FA and NK contributed essential reagents or tools. CN, AB, RM, NG, AK, EK, NS, SH, IR, GZ, CL, DJ, CW, FA and NK analysed the data. CN, NG, AK, EK, NS, SH, IR, GZ, CL, DJ, CW, FA and NK wrote the paper. All authors agreed to submit the paper, and I provided details of all co‐authors.

## FUNDING INFORMATION

The analysis was performed using institutional funds.

## CONFLICT OF INTEREST STATEMENT

The authors declare no conflict of interest.

## ETHICS STATEMENT

The retrospective study was performed according to the Declaration of Helsinki.

## PATIENT CONSENT STATEMENT

Every patient consented to the treatment and to analyse the results.

## PERMISSION TO REPRODUCE MATERIAL FROM OTHER SOURCES

No material from other sources was used.

## CLINICAL TRIAL REGISTRATION

This is a retrospective study without clinical trial registration.

## Supporting information


Data S1.

## Data Availability

Data are available after request to the corresponding author.
