# Supplementary material for: Different clearance of KITD816V mutation and tryptase levels after haematopoietic cell transplantation in patients with systemic mastocytosis with associated haematological neoplasm
Source: Br J Haematol. 2025 Jun 17;207(2):509–14. doi: 10.1111/bjh.20211 (PMC12378900; doi:10.1111/bjh.20211)
Supplement: Supplementary file 1 — Data S1. [file BJH-207-509-s001.pdf]

Appendix

Suppl. References

Ref S1 Lübke, J., et al., *Allogeneic Hematopoietic Cell Transplantation in Advanced Systemic Mastocytosis: A retrospective analysis of the DRST and GREM registries.* Leukemia, 2024. **38**(4): p. 810-821.

Ref S2 Ustun, C., et al., *Hematopoietic stem-cell transplantation for advanced systemic mastocytosis.* J Clin Oncol, 2014. **32**(29): p. 3264-74.

Ref S3 McLornan, D.P., et al., *Allogeneic haematopoietic cell transplantation for advanced systemic mastocytosis: Best practice recommendations on behalf of the EBMT Practice Harmonisation and Guidelines Committee.* Leukemia, 2024. **38**(4): p. 699-711.

Legend to figures:

Figure S1: A: Donor Chimerism versus *KIT* D816V mutation (n=7 patients) pre HCT (blu) and post HCT at 6 months (orange). **B:** Donor chimerism versus tryptase (B; n=7 patients) pre HCT (blu) and post HCT at 6 months (orange)

Figure S2 Patterns of *KIT* D816V mutation, chimerism and tryptase in patient no.7 with CMML-2 showing rejection/relapse 10 months after first HCT (A). Patient received stem cell boost because of pancytopenia and donor lymphocyte infusion (DLI) following decrease of donor chimerism. After preparative regimen with fludarabine (FLU)/TBI 800 cGy a 2. HCT (B) was performed. Fast normalization of tryptase and continuous remission of CMML-2 was observed. Bone marrow (BM) ▲■, peripheral blood (PB) ▲■ ,

Figure S3 Patterns of *KIT* D816V mutation, chimerism and tryptase in patient no.8 with PMF-AML showing molecular relapse 4.5 months and hematological relapse 9.5 months after HCT followed by increase of *JAK2* V617F. Bone marrow (BM) ▲■ , peripheral blood (PB) ▲■

Figure S4 Patterns of *KIT* D816V mutation, chimerism and tryptase in patient no.11 with CMML and a decrease of tryptase followed by a second peak and normalization within 7 months after HCT.

Figure S5 Patterns of *KIT* D816V mutation, chimerism and tryptase in patient no. 9 with AML showing full donor chimerism and a decrease of tryptase with a second peak 3 months and normalization within 24 months after HCT.

Figure S6 Patterns of *KIT* D816V mutation, chimerism and tryptase in patient no.13 with CMML-I with high tryptase levels despite full donor chimerism and *KIT* negativity within one month after HCT, but persistent high tryptase levels till 7 months after HCT. The markedly increased tryptase level normalized after removal of an enlarged spleen infiltrated with 50% *KIT* D816V mutation mast cells. Bone marrow (BM) ▲■ , peripheral blood (PB) ▲■

Figure S7 Patterns of *KIT* D816V mutation, chimerism and tryptase in patient no.12 with CMML with fast full donor chimerism and decrease of *KIT* D816V mutation, a decrease in tryptase within 10 months after HCT followed by an increase thereafter without evidence of systemic mastocytosis. Bone marrow (BM) ▲■ , peripheral blood (PB) ▲■

Figure S8 Patterns of *KIT* D816V mutation, chimerism and tryptase in patient no.10 with CMML-2 with chimerism, disappearance of *KIT* D816V mutation and low tryptase levels. Abbreviations: Bone marrow (BM) ▲■ , peripheral blood (PB) ▲■

Table S1: Characteristics of patients with SM-AHN (n=13) at diagnosis, at and outcome after hematopoietic Cell Transplantation (HCT)

at diagnosis					at HCT											Donor and HCT characteristics				outcome			
n	AHN- disease	cytogenetic	*Diagnosis MC KIT*	Treatment SM	Age (y)/sex	Karnofsky index	disease	stage	cytogenetic	KIT D816V allele burden and other mutations	MC %	Tryptase	diseases phenotype	B / C-findings	MARS	Age (y)/sex	Type/HLA-match	Interval Dg – HCT (y)	Prepar.Regimen; GvHD prophylaxis	Max. Chimerism (%)	SM-AHN outcome	relapse	alive
1	MDS/ MPN	46,XY	**ASM-AHN; MCL-AHN?** SC, CD25+, BM *KIT*D816V, UP; MC+;	no	68/m	100	MDS/ MPN	untr	46,XY	n.a. No NGS	0	n.a.	CD25	SPLMGY (C) 12x4x10		43/f	U; HLA-C≠	1.9	FLAMSA_ BuFlu; CSA/MMF (RIC)	98.9	No other mutations	no	yes
2	MDS/ MPN; CD33^{+},CD64^{+}, CD65^{+},CD15^{+}, CD135^{+},CD38^{+}, CD4^{+},CD56^{+} JAK2^{-}	n.a.	**MCL-AHN** MFDI, CD25^{+}, tryptase 30 µg/L, D816V^{+}, BE >10%, SC CD117^{+}, MC 50% (B) BM CD14^{+}, LN (B), ascites (C) cytopenia(C)	Midostaurin	56/m	80	AML	CR1, LDH 1103	48;XY, +8,+8, 46, XY	*KIT* D816V BM, JAK2^{-} CEBPA^{-}, MLL^{-} other mutations n.a.	1%, infiltration LWK 5/ SWK	1.38	Mono 19%; CD64^{+}, CD13^{+}, CD33^{+}, CD15^{+}, NG2	SPLMGY (C) 21 cm, ascites (C), large osteolysis (C) spine; bone pain		28/m	U;=	3.2	BuCy; TAC/MMF (MAC)	99.9	*KIT*D816V decreasing 0,0% at +23 mts; 46, XY 6-23 mnt, SPLMGY (C), aszites (C), other mutations n.a.	no	yes
3	AML	46,XY	**MCL-AHN** MFDI, MC 80% (B) BM, CD117^{+}	no	74/m	80	AML	PR, 30% blasts	46;XY	MLL^{-}, IDH^{-}, DNMT3A^{-}, FLT3^{-}, NPM1^{-} other mutations n.a.	0 MC	11.9		no		23/m	U;=	0.4	FLAMSA_ Treo; CSA/MMF (RIC)	99.9	*KIT*D816V 0,0% +12-20 mts BM; *KIT* M541L 47,8% +6-20 mts; 46;XY;	no	yes
4	MDS/ MPN; SRSF2 24%, TET2 13%, CMML infiltration skin	46,XY	**MCL-AHN** MFDI, SC 25%, CD25^{+}, D816V 1,2% BM, 0.0% PB, tryptase 24 ug/l, MC <5%, CD117^{+}, UP/anaphy, skin (MC 20%, SC, CD25^{+})	cladribine	57/m	100	MDS/ MPN	PR	46,XY	KIT D816V 0%, TET2 O1425fs* 13.9%	20%, skin infiltrates	18		SPLMGY (C), 12,2x5,5 (C).cytopenia (C)		25/m	R;haplo	3.7	BuFlu other; post Cy (MAC)	99.9	SPLMGY (C), **TET2, O1325fs* 36.5%**	no	yes
5	MDS; TET2^{+}, KRAS^{+}, EZH2^{+}	46,XY /47,XY,+8	**ASM-AHN (MCL-AHN?)** MFDI, CD25^{+}, SC, D816V, MC increased, SPLMGY (C) Skin: cMC, non-LHC Histiozytosis, CD117^{+} MC	Azacytdine	73/m	n.a.	AML	PR 1	46,XY	KIT D816V 0%,; TET2 T779fs* 46.8%; KRAS Y64N 17.5%; T58I 28.7%; EZH2 L671R 24.9%	increased		MFDI, CD25^{+},	cytopenia (C), 3 minor criteria, no SPLMGY		38/f	U; = BM	0.9	FLAMSA/ Treo; CSA/MMF (RIC)	99.9	MC increased (BM) +4 mts; LDH 235, L 3.0, TET2^{-}, **KRAS T58I 5.2%**, EZH2-	yes	no
6	CML	46,XY t(9;22) (q34;q11)	**ASM-AHN** MFDI, SC, CD25^{+}, KIT D816V 0.06%, tryptase 10.7, MC 1,5%, PA, SI, PV	Dasatinib, Ponatinib	58/m	60	CML	AP 2	46,XY t(9;22) (q34;q11)	n.a. SF3B1 D621N 18.8% DNMT3A R736H 50.7%	20%	6.35		Cytopenia (C)		55/m	U;=	1.6	TreoFlu; CSA/MMF (RIC)	99.9	MR, CR after DLI MC 0% (BM), 46;XY; LDH 273; L 4.1; mutations n.a.	MR/ CR	yes
7	CMML-2; ASXL1^{+}, DNMT3A^{-}, SRSF2^{-}	46,XY	**MCL-AHN** MFDI, SC, KIT D816V 46%, K558E 28.6%, SPLMGY 22x9 cm	no	53/m	80	MDS/ MPN	PD	46;XY	KIT D816V 46%; RUNX1 P125fs* 45.4%; KRAS Q61R 2.9%	20	23.4	MFDI, SC,	massive SPLMGY (C), cytopenia (C)		41/m	U; HLA-DQ≠	1.0	FLAMSA; CSA/MM (RIC)	99.9	MC 80 %, LDH 263, blasts 5% BM, CMML-2 BM, D816V 20.7%; K558E 22.1%; RUNX1 P125fs* 18.7%; KRAS- splenectomy CMML h; rejection and relapse	Yes	yes

7a			**MCL-AHN**<br>See 7	no		n.a				*KIT* D816V 23.4%; see post HCT	20%	106	MFD, SC, CD25, MC 20%,CD117+	splenectomy		27/m	U;=	1.9	TBI (8Gy)Flu; CSA/MMF (MAC)	99.9	Mutation n.a.	no	yes
8	PMF; *JAK2+ RUNX1+*	complex	**MCL-AHN**<br>*MFDI (M), KITD816V 0,12%; atypias, CD 25+, CD 117+ , MC-30%,*	*AraC/Dauno, ladribine AraC/Dauno*	60/m	100	AML	PD	complex	KIT D816V 0.1%, RUNX1 23,7%, JAK2 48,0%, FLT3-, IDH-, NPM1-, No NGS	15%	14.1	SC, CD117+	SPLMGY (13 cm)		29/m	U;=	3.0	BuFlu; CSA/MMF (MAC)	99.9	no mutations BM at 3 mts; AML MR at +4 mts, HR +9 mts; MC 5% (h); PB: IDH1R132H 6,9%; GATA2 W360R 8,3%; JAK2 V617F 28,3%, NF1F103fs*7 41,2% ZRSR2S447-R448dup 57,8%; LDH 172, L 6.2, blasts >5 %	yes	no
9	MDS /MPN<br>RUNX1+ ASXL1+ U2AF1+	47, XY,+11	**MCL-AHN**<br>MC 80%, skin: cMC osteolysis	no	83/m	80	AML	untr	n.a.	KIT D816V 40,2%, RUNX1 T178I 4.6% U2AF1 S34F 44.6% ASXL1 A761T 48.9%	45	>200	Atypic SC	No SPLMGY		52/m	R; haplo	0.3	BuFlu other; post Cy (RIC)	99.9	MC 30%, CD25+, CD117+, SC, tryptase+ at **+3 mts (b)**, MC 15% CD25+, CD117+ SC CD117+ at **+5 mts;** MC <5%, SC at **+7.5 mts** (b) MC 10% CD25+, CD117;Myeloid Panel negativ**+13 mts** (b), **24 mts** (b)	no	yes
10	MDS/ MPN JAK2+ 4.9%	46,XX	**ASM-AHN**<br>*KIT*D816V 15%, tryptase 27, CD25+, MC <20%, Eaching, food allergy, UP	no	58/f	80	MDS/ MPN	untr	46, XX	Aberr MC 5%; KIT D816V 9.9%, TET2 798* 98.1%	8	26,9	SC CD25+ CD30+	SPLMGY (C; 17,3x4,3)		62/m	R;=	2.8	BuTT; TAC/MMF (MAC)	99.9	MC 0% BM (h) at **+2/+7 mts** MC increased (c) **DNMT3A R729W** 15.3%; 46 XY **+2/+4/+7/+10 mts;** JAK2- 10 mts	no	yes
11	MDS/ MPN SRSF2 46,2%, TET2 49%, ASXL 143,3%	n.a.	**MCL-AHN**<br>MC 50%, *KIT* D816V LN, 117+ ,tryptase+, Pruritus, Splenomegaly (C), Lymphadenopathie (B),	no	61/f	80	MDS/ MPN	untr	46;XX	KIT D816V 0.06% PB SRSF2 P95H 54.3%, TET2 S348fs* 48.6%	50, 0%	136	CD117	smoldering SM (B); SPLMGY (C; 17,3x4,3); Elevated LFP (C)	high	34/f	R;haplo	3.6	BuFlu other; post-Cy (RIC)	99.9	46;XX +6 mts MC 10% CMML 0%, liver cirrhosis spleen 17,5 cm +7 mts MC 0% FACS, BM, spontaneous bacterial peritonitis neg myeloid panel (ASXL1-, SRSF2 P95H-, TET2 S348fs*-)	no	yes
12	MDS/ MPN CD117+, ASXL1+,U2AF+, BRAF+, RUNX+	46,XY	**MCL-AHN**<br>MFDI, SC, CD25+, *KIT*D816V, tryptase MC70%,	Midostaurin pre and post HCT	73/m	80	MDS/ MPN	NR	46,XY	KIT D816V 42.5/47.2%, BRAF G32fs*22 14.9%, RUNX1 D62fs*76 38.5%, U2AF1 Q157P 41.8%	MC 35% (c);70% (h)	>200		tryptase>200 (B); SPLMGY (C); cytopenia (C)	high	23/f	U; HLA-A Ag≠	0.5	TreoFlu; CSA/MMF (RIC)	99.9	massive MC+1mts cytomorpho; +4 mts MC 70% (h), +7 mts MC 60% +11 mts MC 20%, atyp. MC; 15 mnt 0%; CMML CR, +4/+2/+11 mts ASXL1-, U2AF1-, BRAF-, RUNX1-,**CALR E398D 52.2%**	no	yes
13	MDS/ MPN	n.a.	**MCL-AHN**<br>MFDI, *KIT*D816V (PB 39,9%, BM 87,9%)SC atypic, CD117+, CD25+, CD30+, MC 30%, weight loss (C)	Midostaurin Ruxolitinib	60/m	80	MDS/ MPN	n.a.	n.a.	KIT D816V 39,9/43.4% SRSF2 P95L 57,6% RUNX1 54,4%	70%	>200		SPLMGY (C 17x6 cm); Lymphadenopathy (B), cytopenia (C)	high	46/m	U;=	0.4	BuTT; CSA/MMF (MAC)	99.9	+2/+6 MC 90% BM, MC liver and spleen MC 50%, KITD816V 11.3%, CD25+ ,CD123+,CD117+ No mutation 16 mts after HCT	no,	yes
	median (range)				60.0 (53-83)	80 (60-100)										34 (23-62)		1.9 (0.4-3.7)					

Abbreviatons: =, HLA identical; anaphhy, anaphlaxia; AraC, cytosine arabinoside; b, biopsy; BE, blast excess; (B) B findings; c, cytology; (C) findings; cMC, CD, cluster differentiation; cutaneous Mastocytosis; h, histology; haplo, haplotype compatible donor; HLC, Langerhans cell; HR, hematological relapse; L, leucocytes 10^9/l;LN, lymphnode; (M), major criteria; (m), minor criteria; MC, mast cells; MFDI, multi focal dense infiltrates of MC in BM or extracutan organs; MR, molecular relapse; mts, months; PA, Psoriasis arthritis; PR, partial remission; PV, Psoriasis vulgaris; SC, spindelcell; SI, Sacroiliitis; SM, systemic mastocytosis; SPLMGY, splenomegaly; untr, untreated; UP, urticaria pigmentosa;

;

Table S2: Outcome and relapse of AHN according to diagnosis, type of SM and preparative regimens

		AHN-relapse			alive		
	n	n	%	p-value*	n	%	p-value*
SM-AML	5	2	40.0	n.s.	3	60.0	0.09
SM non-AML	8	2	25.0		8	100.0	
Aggressive systemic mastocytosis (ASM)-AHN	4	2	50.0	n.s.	3	75.0	n.s.
Mast Cell Leukemia (MCL)-AHN	9	2	22.2		8	88.9	
Reduced intensity conditioning (RIC)	8	3	37.5	n.s.	7	87.5	n.s.
Myeloablative conditioing (MAC)	6	1	16.7		5	83.3	

- Fisher´s exact test

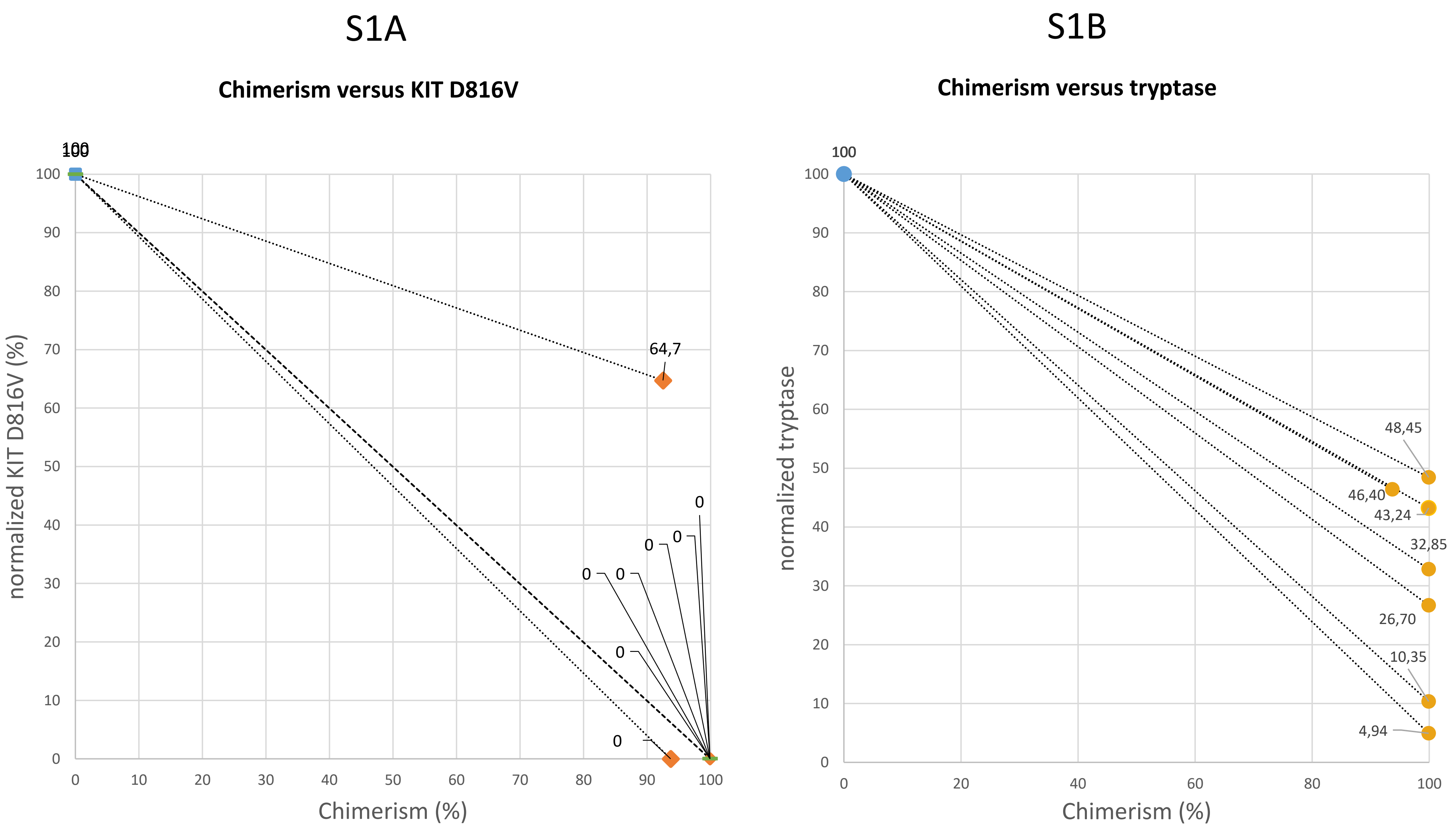
S1A
Chimerism versus KIT D816V
normalized KIT D816V (%)
Chimerism (%)
100
64,7
0
S1B
Chimerism versus tryptase
normalized tryptase
Chimerism (%)
100
48,45
46,40
43,24
32,85
26,70
10,35
4,94

Figure S2

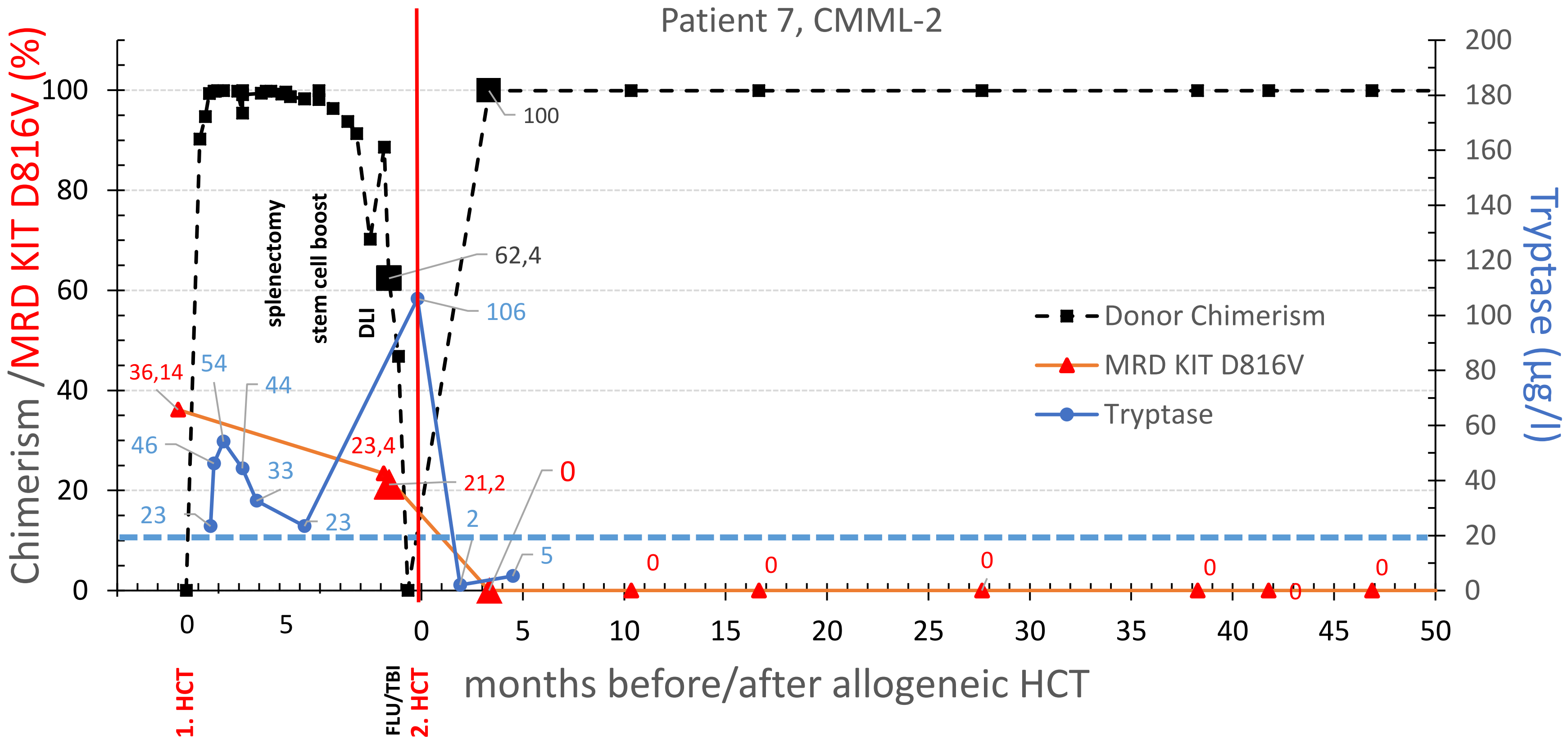

Patient 7, CMML-2
Chimerism /MRD KIT D816V (%)
Tryptase (µg/l)
months before/after allogeneic HCT
1. HCT
2. HCT
FLU/TBI
splenectomy
stem cell boost
DLI
Donor Chimerism
MRD KIT D816V
Tryptase
36,14
23,4
21,2
62,4
100
106
54
46
44
33
23
23
2
5
0

Figure S3

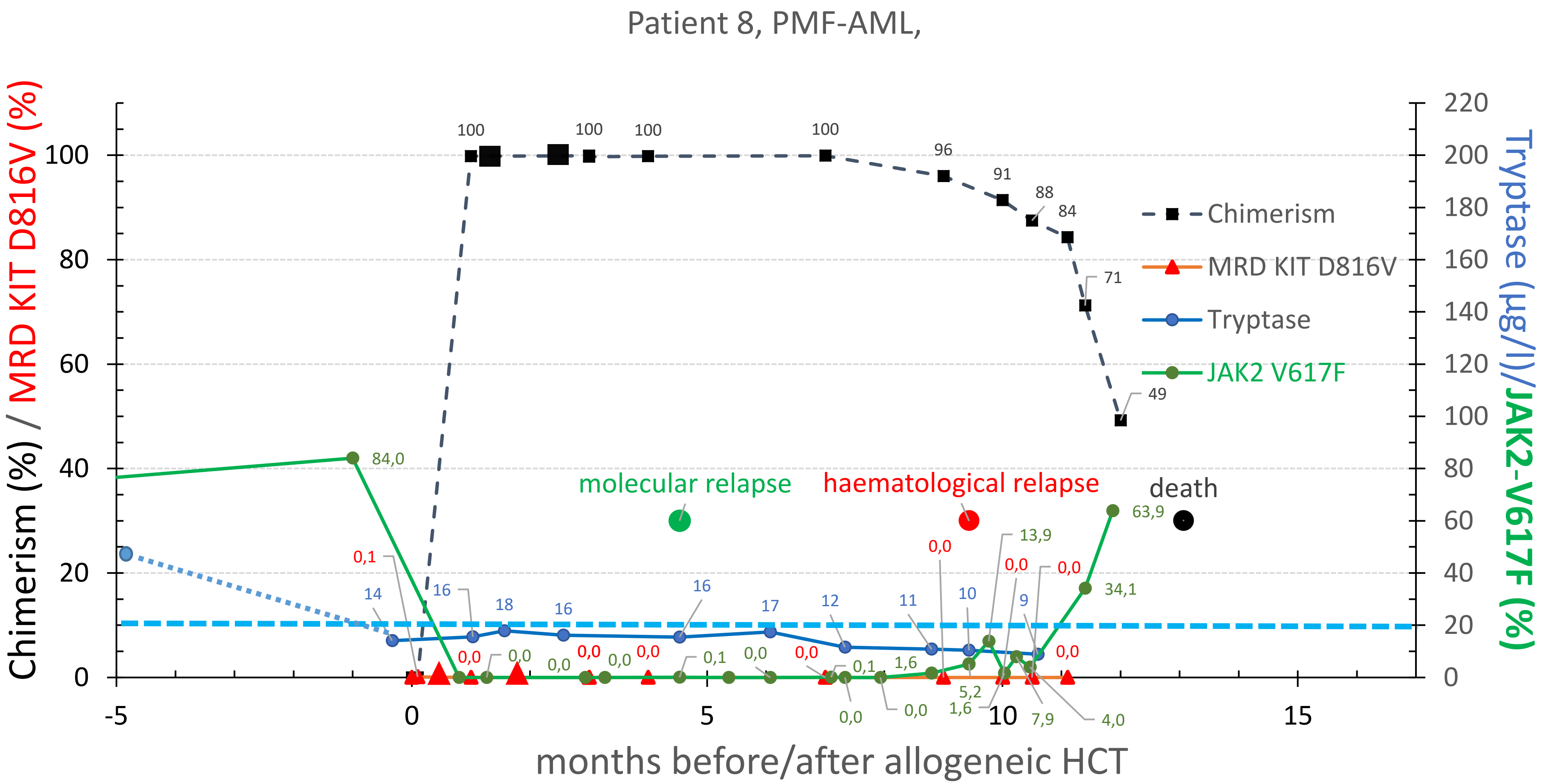

Patient 8, PMF-AML,
Chimerism (%) / MRD KIT D816V (%)
Tryptase (µg/l)/JAK2-V617F (%)
months before/after allogeneic HCT
Chimerism
MRD KIT D816V
Tryptase
JAK2 V617F
molecular relapse
haematological relapse
death

Figure S4

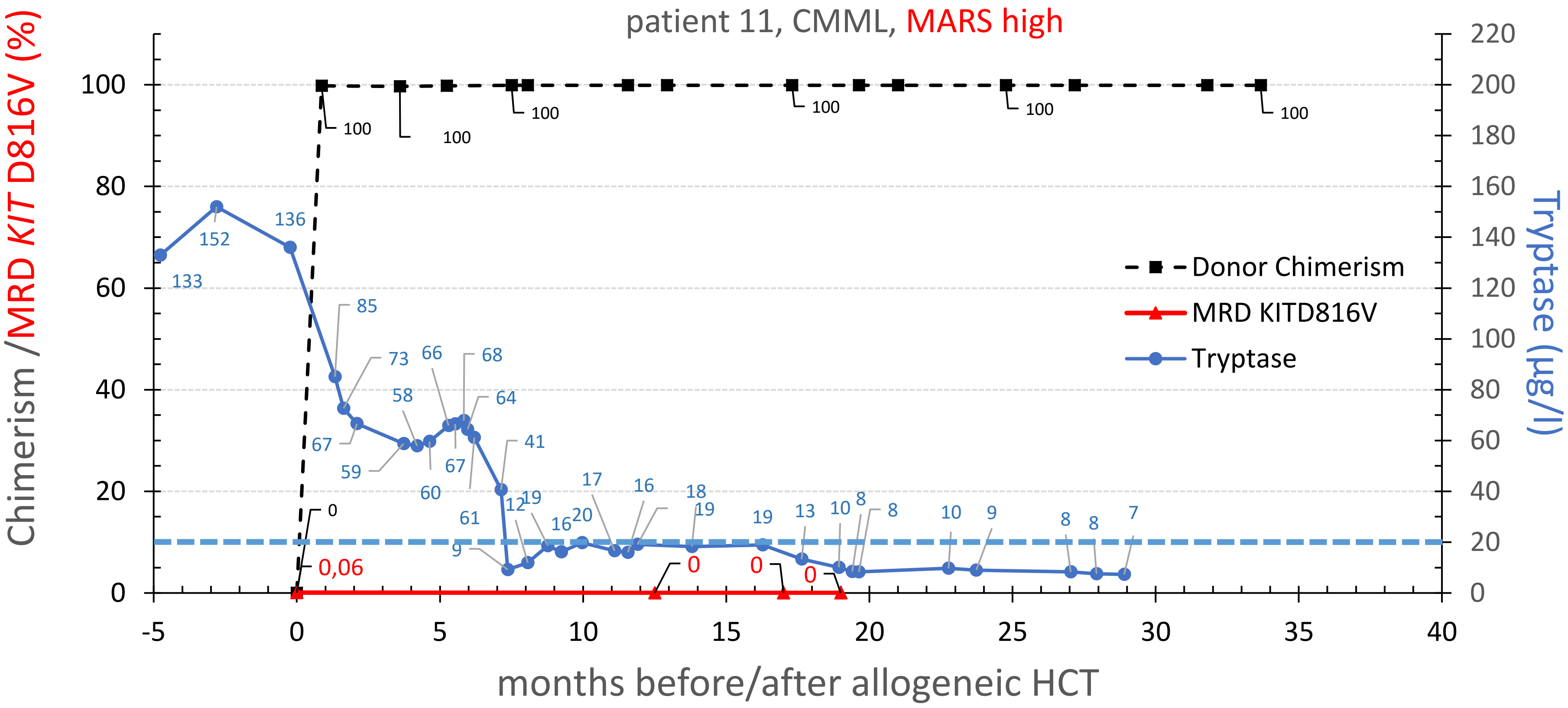

patient 11, CMML, MARS high
Chimerism /MRD KIT D816V (%)
Tryptase (µg/l)
months before/after allogeneic HCT
Donor Chimerism
MRD KITD816V
Tryptase
0,06

Figure S5

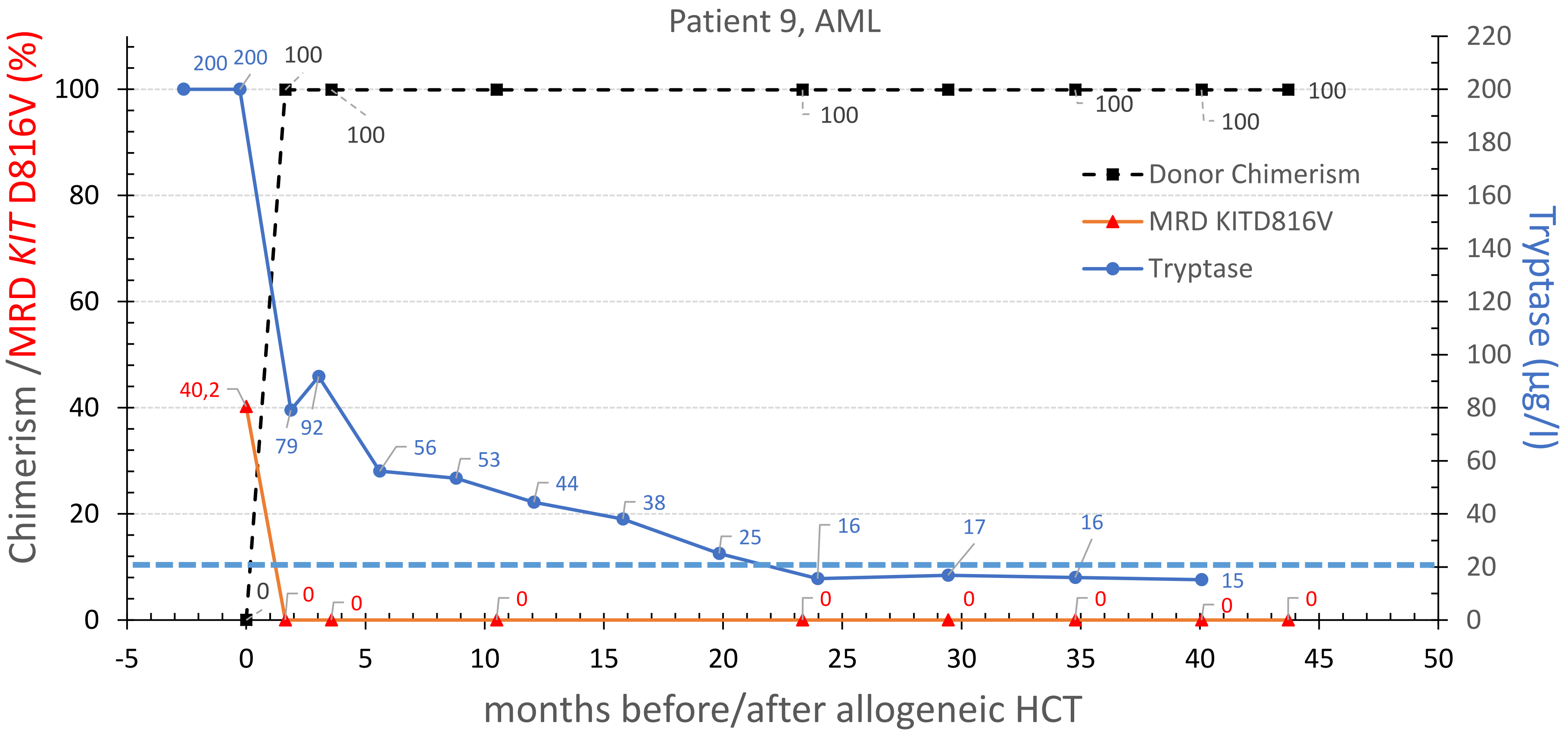

Patient 9, AML
Chimerism /MRD KIT D816V (%)
Tryptase (µg/l)
months before/after allogeneic HCT
Donor Chimerism
MRD KITD816V
Tryptase
200
200
100
100
100
100
100
100
40,2
79
92
56
53
44
38
25
16
17
16
15
0
0
0
0
0
0
0
0
0
0

Figure S6

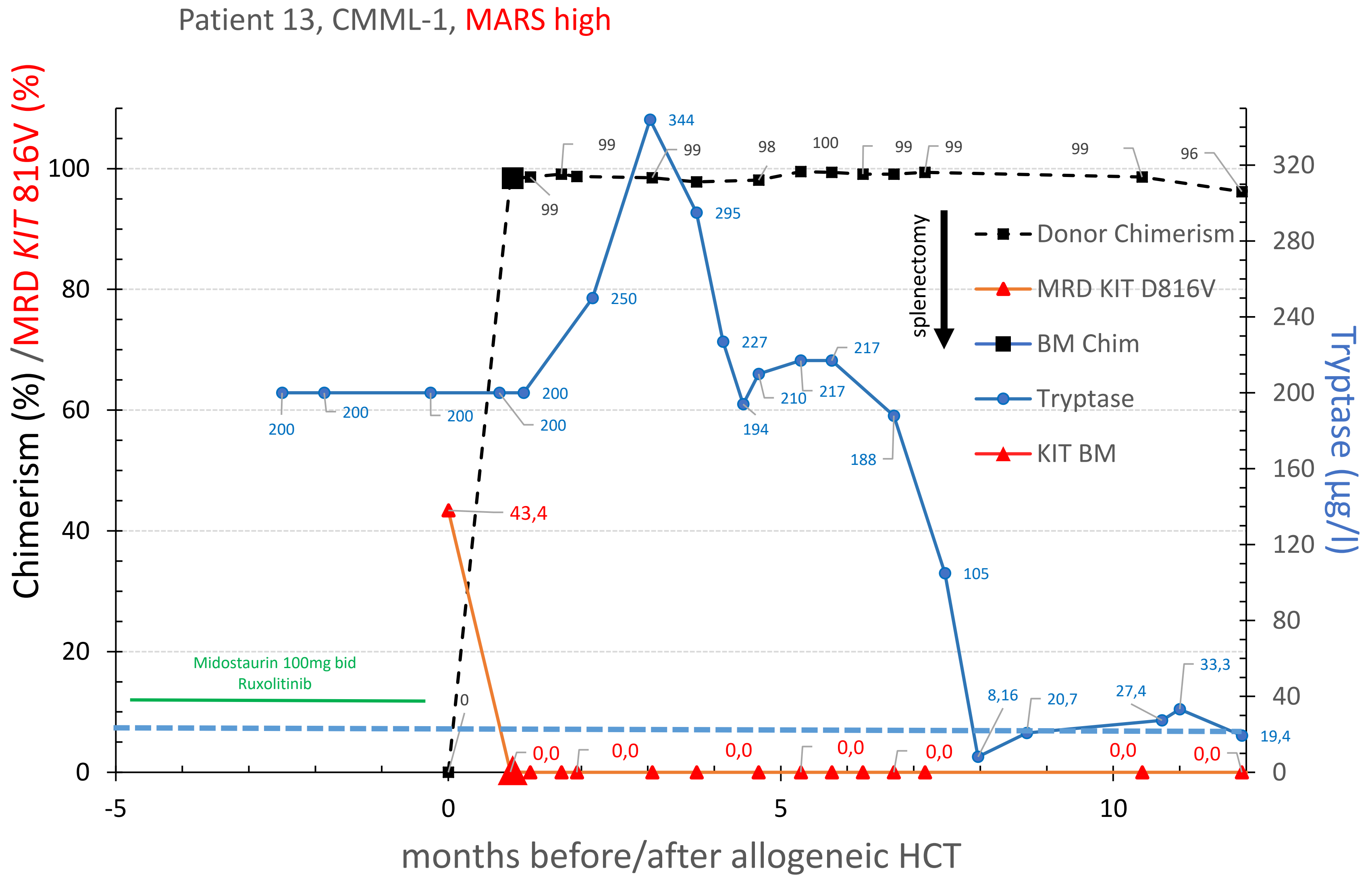
Patient 13, CMML-1, MARS high
Chimerism (%) /MRD KIT 816V (%)
Tryptase (µg/l)
months before/after allogeneic HCT
Donor Chimerism
MRD KIT D816V
BM Chim
Tryptase
KIT BM
splenectomy
Midostaurin 100mg bid
Ruxolitinib
43,4
0,0
344
295
250
227
217
210
194
188
105
8,16
20,7
27,4
33,3
19,4
200
99
98
100
96

Figure S7

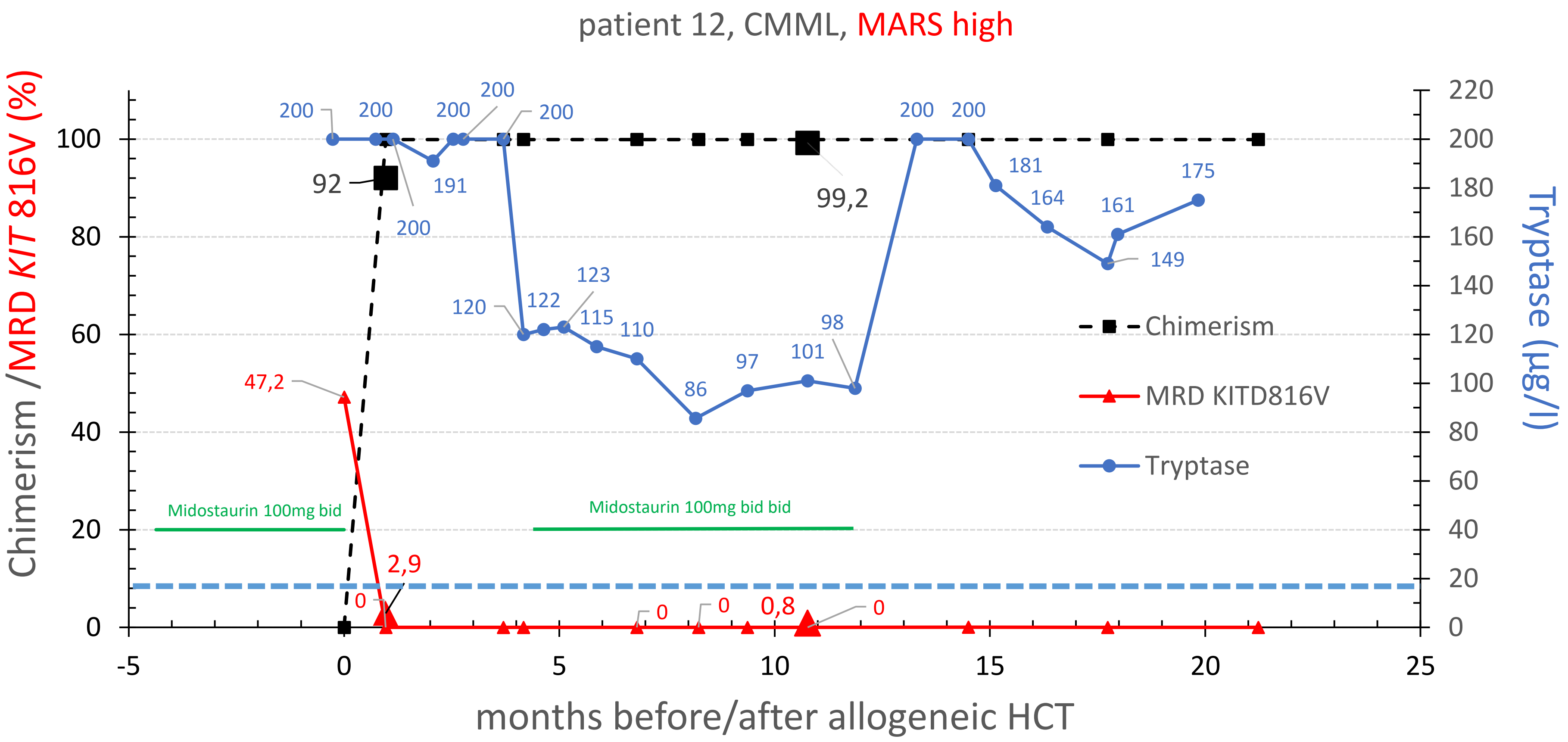
patient 12, CMML, MARS high
Chimerism /MRD KIT 816V (%)
Tryptase (µg/l)
months before/after allogeneic HCT
Midostaurin 100mg bid
Midostaurin 100mg bid bid
Chimerism
MRD KITD816V
Tryptase
92
99,2
47,2
2,9
0,8

Figure S8

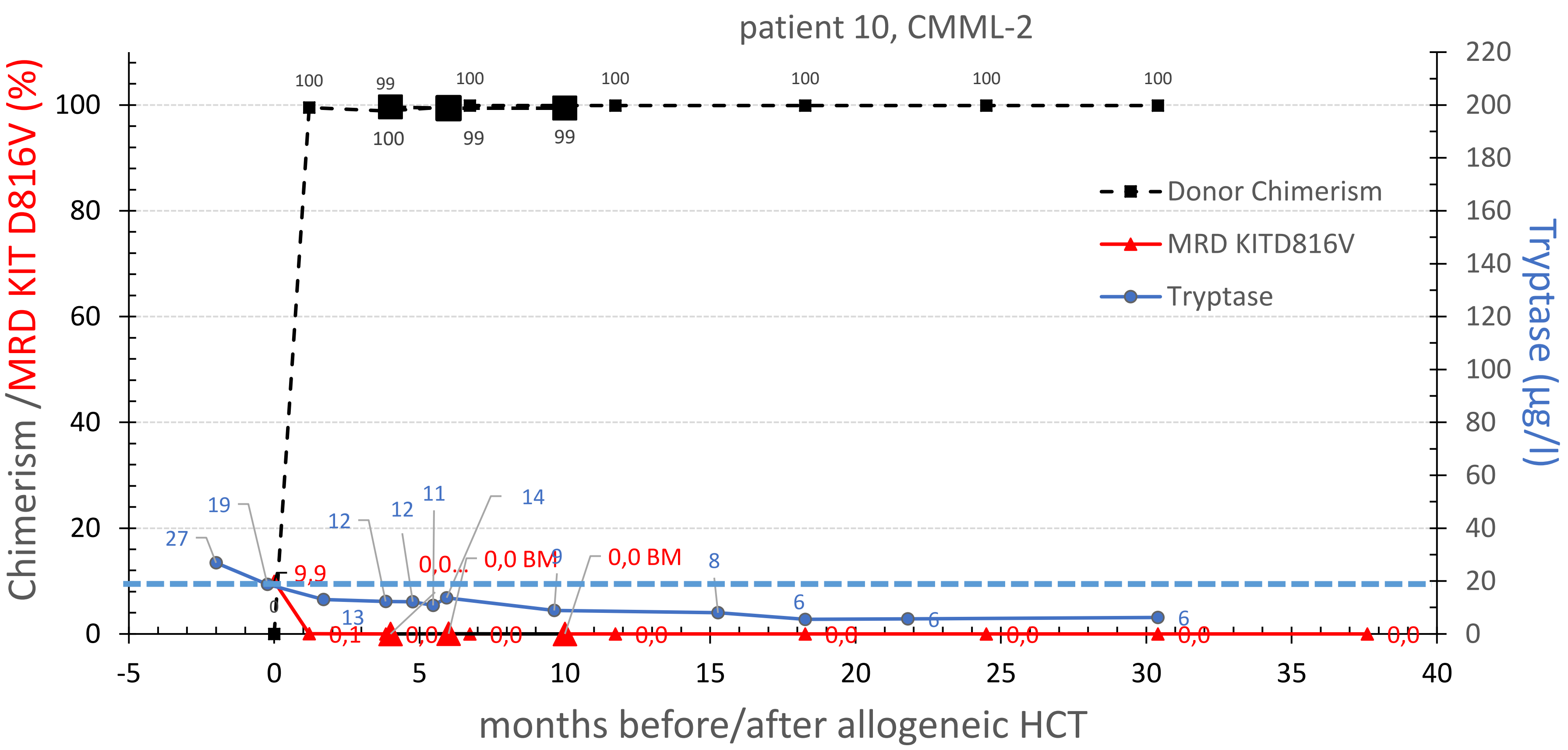

patient 10, CMML-2
Chimerism /MRD KIT D816V (%)
Tryptase (µg/l)
months before/after allogeneic HCT
Donor Chimerism
MRD KITD816V
Tryptase
0,0 BM
0,0 BM
9,9
